# Study protocol: randomized phase III trial of neo-adjuvant and adjuvant chemotherapy vs. immediate surgery and adjuvant chemotherapy for localized soft tissue sarcoma: Japan Clinical Oncology Group study JCOG2102 (NACLESS)

**DOI:** 10.1093/jjco/hyae160

**Published:** 2024-11-05

**Authors:** Yuki Funauchi, Satoshi Tsukushi, Hiroaki Hiraga, Akio Sakamoto, Toshiyuki Kunisada, Akihito Nagano, Koji Hiraoka, Kazutaka Kikuta, Tsukasa Yonemoto, Keisuke Ae, Akira Kawai, Makoto Endo, Yusuke Sano, Ryunosuke Machida, Tetsuya Sekita, Haruhiko Fukuda, Yoshinao Oda, Toshifumi Ozaki, Kazuhiro Tanaka, JCOG Bone Soft Tissue Tumor Study Group

**Affiliations:** Department of Orthopaedic Surgery, Tokyo Medical and Dental University (TMDU), Tokyo, Japan; Department of Orthopaedic Surgery, Cancer Institute Hospital of Japanese Foundation for Cancer Research, Tokyo, Japan; Division of Orthopaedic Surgery, Aichi Cancer Center Hospital, Nagoya, Japan; Department of Musculoskeletal Oncology, National Hospital Organization Hokkaido Cancer Center, Sapporo, Japan; Department of Orthopaedic Surgery, Graduate School of Medicine, Kyoto University, Kyoto, Japan; Department of Orthopaedic Surgery, Okayama University Graduate School of Medicine, Dentistry, and Pharmaceutical Sciences, Okayama, Japan; Department of Orthopaedic Surgery, Gifu University Graduate School of Medicine, Gifu, Japan; Department of Orthopedic Surgery, Kurume University School of Medicine, Kurume City, Fukuoka, Japan; Division of Musculoskeletal Oncology and Orthopaedic Surgery, Tochigi Cancer Center, Tochigi, Japan; Division of Orthopaedic Surgery, Chiba Cancer Center, Chiba, Japan; Department of Orthopaedic Surgery, Cancer Institute Hospital of Japanese Foundation for Cancer Research, Tokyo, Japan; Division of Musculoskeletal Oncology and Rehabilitation Medicine, National Cancer Center Hospital, Tokyo, Japan; Department of Orthopaedic Surgery, Kyushu University, Fukuoka, Japan; Japan Clinical Oncology Group Data Center/Operations Office, National Cancer Center Hospital, Tokyo, Japan; Japan Clinical Oncology Group Data Center/Operations Office, National Cancer Center Hospital, Tokyo, Japan; Japan Clinical Oncology Group Data Center/Operations Office, National Cancer Center Hospital, Tokyo, Japan; Japan Clinical Oncology Group Data Center/Operations Office, National Cancer Center Hospital, Tokyo, Japan; Department of Anatomic Pathology, Graduate School of Medical Sciences, Kyushu University, Fukuoka, Japan; Department of Orthopaedic Surgery, Graduate School of Medicine, Kyoto University, Kyoto, Japan; Department of Advanced Medical Sciences, Oita University Faculty of Medicine, Oita, Japan

**Keywords:** soft tissue sarcoma, randomized controlled trial, surgery, neoadjuvant chemotherapy, adjuvant chemotherapy

## Abstract

The optimal timing of surgery and the number of courses of perioperative chemotherapy for high-risk soft tissue sarcoma patients are still controversial. Tumour growth during neoadjuvant chemotherapy led to limb amputation in some patients. This study aims to confirm the non-inferiority of surgery and three courses of adjuvant chemotherapy with adriamycin (30 mg/m^2^, days 1 and 2) plus ifosfamide (2 g/m^2^, days 1–5) compared with our standard treatment of three courses of neoadjuvant chemotherapy and surgery followed by two courses of adjuvant chemotherapy with adriamycin plus ifosfamide for localized high-risk soft tissue sarcoma patients. This is a multi-center, two-arm, open-label, randomized phase III trial. The primary aim is to confirm the non-inferiority in overall survival (margin: hazard ratio of 1.61). This is the first randomized controlled trial to compare neoadjuvant chemotherapy and immediate surgery for soft tissue sarcoma. This trial was initiated on 16 November 2022 and registered with the Japan Clinical Trials Registry (jRCTs031220446).

## Teaser

The Neo-adjuvant and adjuvant chemotherapy vs. Adjuvant Chemotherapy for LocalizEd Soft tissue Sarcoma study is a phase III trial comparing the non-inferiority of immediate surgery with adjuvant chemotherapy versus neoadjuvant chemotherapy and surgery for high-risk soft tissue sarcoma, focusing on overall survival.

## Introduction

Soft tissue sarcomas (STSs) are rare malignant tumours of mesenchymal origin that arise from all body soft tissues, including fibrous, adipose, muscular and vascular tissues. It accounts for ~1% of all malignant tumours and is one of the most common rare cancers. According to the SEER (Surveillance Epidemiology and End Results) Database, STS accounted for 48 012 (1.5%) of the 3.11 million malignancies registered in the USA (1).

The standard treatment of stage I and II non-round cell STS is surgery alone. The 5-year survival proportion is ~95% for stage I and ~80% for stage II (2, 3). For stage III cases with superficial lesions, surgery alone is still considered the standard treatment since the 5-year survival proportion is ~70% or more. On the other hand, the 5-year survival proportion for stage III patients with deep lesions, which are located deeper than the superficial fascia, is poor at ~50% if treated with surgery alone, so perioperative chemotherapy is often used in addition to surgery (2, 4, 5). In recent clinical trials, perioperative chemotherapy with adriamycin (ADM) or other anthracycline, plus ifosfamide (IFO), has been employed for ‘high-risk’ cases, such as high-grade, >5 cm in diameter and deep-seated (6–10). A meta-analysis of 18 trials revealed a significant increase in overall survival (OS) with ADM plus IFO (AI)–based perioperative chemotherapy (11). So far, there are 10 papers on clinical trials of perioperative chemotherapy with AI regimen, although the doses, timing of administration and number of courses have not been standardized (Table 1).

**Table 1:** Summary of clinical trials of perioperative adjuvant chemotherapy with adriamycin plus ifosfamide for localized soft tissue sarcoma

Author	Year	Study type	*N*	5y-OS	5y-PFS	NAC/AC	Dose	Course
JCOG0304 (6)	2015	Single arm	70	82.6	63.8	NAC + AC	A 300/I 50	5(3 + 2)
JCOG1306 (7)	2022	RCT	70	91.4^a^	73.5^a^	NAC + AC	A 300/I 50	5(3 + 2)
Gortzak E (12)	2001	RCT	67	65	56	NAC	A 150/I 15	3
Gronchi A (8)	2012	RCT	161	71	61.1	NAC + AC	E 600/I 45	5(3 + 2)
			160	68	61.4	NAC	E 360/I 27	3
Gronchi A (9)	2017	RCT	144	89^a^	62^a^	NAC	E 360/I 27	3
Brodowicz T (13)	2000	RCT	31	88.1	70.8	AC	A 300/I 36	6
Frustaci S (4)	2001	RCT	53	69^a^	50^a^	AC	E 300/I 45	5
Petrioli R (14)	2002	RCT	45	72	69	AC	E 300/I 24	4
Woll PJ (10)	2012	RCT	175	67.8	54.9	AC	E 375/I 25	5
Schliemann C (15)	2018	Single arm	104	75.6	61.2	AC	A 300/ I 20	4

RCT, randomized controlled trial; OS, overall survival; PFS, progression-free survival; A, ADM (mg); I, IFO (g); E, epirubicin (mg).

^a^Percentage of survival at 46–48 months.

The Bone and Soft Tissue Tumor Study Group (BSTTSG) of the Japan Clinical Oncology Group (JCOG) conducted JCOG0304 and the subsequent JCOG1306 trials in Japan to establish the standard treatment for stage III high-risk STS patients. JCOG0304 evaluated the efficacy and safety of a treatment regimen of pre-operative and post-operative AI. That trial showed a good 5-year OS of 82.6% and 5-year progression-free survival (PFS) of 63.8% in patients at high risk (6), establishing three courses of neoadjuvant chemotherapy (NAC) followed by surgery and two courses of adjuvant chemotherapy (AC) with AI as the standard treatment. However, the primary tumour became a progressive disease (PD) according to the revised Response Evaluation Criteria in Solid Tumours (RECIST) guidelines during NAC in 4.3%, and 21% of patients discontinued chemotherapy due to toxicity or patient refusal. This result highlighted the risk of PD during NAC and their high toxicity. In this context, a phase III trial of AI versus gemcitabine plus docetaxel (GD) (JCOG1306) was conducted to confirm the non-inferiority of GD against AI. That trial failed to show the non-inferiority of GD against AI (7). Consequently, three courses of NAC with AI followed by surgery and two courses of AC with AI are considered the standard treatment for stage III high-risk STS patients. That trial also revealed that only 1.4% of the patients showed partial response (PR), whereas 8.6% presented PD during NAC, according to the RECIST guidelines (6,7). As a result, of the 65 patients deemed eligible for limb-sparing surgery at study enrolment, three (4.6%) underwent limb-amputation surgery after NAC. In these cases, limb sparing was possible if surgery was immediately performed after enrolment. Thus, the study revealed the potential disadvantage of NAC: it may cause unnecessary amputation for some patients. Immediate surgery followed by AC may avoid the risk of extended resection due to tumour growth in cases where chemotherapy is ineffective.

Toxicity with AI treatment is also a concern. The most common adverse events are hematologic toxicity, with grade 3/4 leucopoenia and neutropenia almost inevitable, and grade 3/4 non-hematologic toxicity with nausea and anorexia is also common. Given the severe toxicity, a randomized controlled trial (RCT) of three courses of NAC followed by surgery and two courses of AC versus three courses of NAC followed by surgery was recently conducted in Europe (8). The results showed that the 5-year OS was 71% (95% CI: 63–77%) in the former arm compared with 68% (95% CI: 60–75%) in the latter arm, with a hazard ratio (HR) of 1.00 (90% CI: 0.72–1.39), proving non-inferiority in OS. Regarding toxicity, renal dysfunction occurred in 1.5% of patients receiving five courses of chemotherapy but not in those receiving three courses. The incidence of hematologic toxicity also increased with two additional courses, again suggesting that an increase in the number of courses is associated with an increase in toxicity. However, the rationale for using three courses of NAC as the test treatment was not stated in that paper. Globally, there is no evidence of whether chemotherapy or surgery should be performed first. Surgery after NAC is the standard treatment in some countries including Japan and Italy, but other countries and physicians consider resection as the first treatment, with chemotherapy as an option or post-operative consideration. Indeed, in a recently reported clinical trial in Germany for STS, resection was followed by chemotherapy (15).

The National Comprehensive Cancer Network (NCCN) guidelines make no recommendations regarding optimal resection timing or number of courses since there is no evidence (16). The ESMO Clinical Practice Guidelines state that perioperative adjuvant chemotherapy for STSs is a treatment option for high-risk patients. They also mention that three courses of NAC with AI may be effective but do not address the superiority of NAC over AC (17).

The current clinical challenge in this field is to optimize the balance between the survival benefit of perioperative chemotherapy with AI and the toxicity and risk of extended surgery. From this viewpoint, the immediate surgery followed by three courses of AC is expected to be an effective and safer treatment since it will avoid the risk of extended resection due to tumour growth during NAC and reduce the number of chemotherapy courses. We, therefore, designed a randomized phase III trial of Neo-adjuvant and adjuvant chemotherapy vs. Adjuvant Chemotherapy for LocalizEd Soft tissue Sarcoma (NACLESS trial) to confirm the non-inferiority of three courses of AC with AI compared with three courses of NAC and two courses of AC with AI for localized high-risk STS patients for establishing a better standard treatment.

## Patients and Methods

### Study design

This is a multi-centre, randomized, open-label, parallel-arm, phase III trial. Initially, this trial is conducted at 37 centres in Japan, all of which are participating in the JCOG BSTTSG. This trial aims to confirm non-inferiority in OS of immediate surgery followed by three courses of AC compared with the standard treatment of three courses of NAC plus surgery plus two courses of AC in patients with high-risk localized STS. Patients who meet all the following eligibility criteria and none of the exclusion criteria are eligible for enrolment (Table 2). The study protocol was approved by the National Cancer Center Hospital Certified Review Board for Clinical Trials (Certification No. CRB3180008) before initiating patient accrual.

**Table 2:** Summary of inclusion and exclusion criteria of JCOG2102

Inclusion criteria	Exclusion criteria
(1) Grade 2 or 3 (FNCLCC histological grading system) non-round cell soft-tissue sarcoma with histology of undifferentiated sarcoma (pleomorphic, spindle cell), adult fibrosarcoma, myxofibrosarcoma, leiomyosarcoma, synovial sarcoma, liposarcoma (dedifferentiated, myxoid, pleomorphic), pleomorphic rhabdomyosarcoma, malignant peripheral nerve sheath tumour, angiosarcoma (WHO classification 2020) diagnosed by open biopsy specimen (2) 10 or more unstained tumour tissue slides are available (3) Primary tumour (4) Tumour in the extremities or trunk (5) T2-4N0M0 (UICC/AJCC, 8th edition) and deeply seated (localized deeper than the investing fascia, invades the investing fascia or penetrates the investing fascia) according to the latest imaging studies (6) Having measurable lesion on MRI axial section (7) Resectable with a marginal or wide margin (8) Age between 16 and 70 years (9) ECOG performance status (PS) of 0–1 (10) No history of chemotherapy nor radiation therapy for any cancer including non-round cell STS (11) All of the following are met for the most recent laboratory findings within 14 days prior to enrolment: (i) Neutrophil count ≥1500/mm^3^ (ii) Haemoglobin ≥8.0 g/dl (No blood transfusion within 14 days prior to the test used for registration) (iii) Platelet count ≥100 000/mm^3^ (iv) Total bilirubin ≤1.5 mg/dl (v) AST ≤ 100 U/L (vi) ALT ≤100 U/L (vii) Serum creatinine ≤1.5 mg/dl (viii) Creatinine clearance ≥60 ml/min (12) The latest ECG within 35 days prior to enrolment shows normal or no changes requiring treatment (13) Written informed consent	(1) Synchronous or metachronous (within 5 years) malignancies except cancer with 5-year relative survival proportion of 95% or more (2) Active infection requiring systemic therapy (3) Febrile more than 38°C (4) Women (possibly) in pregnancy, within 28 days post-partum, or breastfeeding. Men who wish to get their partner pregnant (5) Patients with psychiatric disorders or psychiatric symptoms (6) Patients requiring systemic steroid medication (7) Poorly controlled diabetes mellitus or routine administration of insulin (8) Poorly controlled hypertension (9) Unstable angina within 3 weeks or with a history of myocardial infarction (10) Poorly controlled valvular disease, dilated cardiomyopathy or hypertrophic cardiomyopathy (11) Positive HBs antigen

FNCLCC, Fédération Nationale des Centre de Lutte Contre le Cancer; ECOG, Eastern Cooperative Oncology Group; ECG, electrocardiogram.

### Interventions

**Figure 1:**
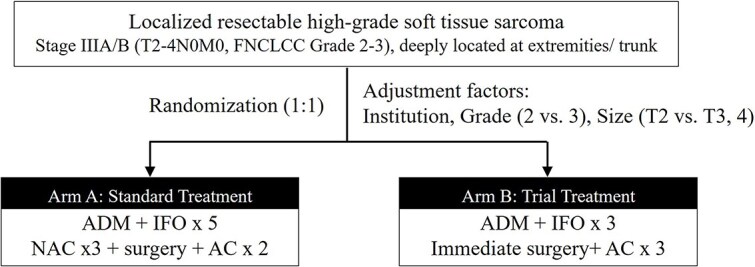
Flowchart of JCOG2102 (Neo-adjuvant and adjuvant chemotherapy vs. Adjuvant Chemotherapy for LocalizEd Soft tissue Sarcoma) study.

Patients will be randomized to arm A or B (Fig. 1) at the JCOG Data Center. Arm A will receive standard treatment (three courses of NAC, surgery and two of AC) and arm B will receive trial treatment (surgery and three courses of AC), with AI comprising adriamycin (30 mg/m^2^, days 1, 2) and ifosfamide (2 g/m^2^, days 1–5).

Random allocation will be based on a minimization method that adjusts for (i) the centre, (ii) histological grade (Grade 2 vs. Grade 3), and (iii) T-classification (T2 vs. T3–4). Detailed procedures for random allocation are not given to participating institutes and investigators.

### Outcomes

The primary endpoint is OS, defined as the period from the date of enrolment until the date of death from any cause. Secondary endpoints are PFS, the response rate of NAC, the pathological response rate of NAC, the proportion of reduction surgery, the proportion of extended surgery, post-operative limb function (extremities), the proportion of limb salvage, adverse events and serious adverse events. PFS is defined as the period from the date of enrolment until the date of progression or death from any cause, whichever is earlier. Progression in this trial includes both PD according to the revised Response Evaluation Criteria in Solid Tumours (RECIST) guidelines and clinical disease progression not confirmed by imaging studies. The response rate of NAC is defined as the proportion of all eligible patients in arm A who are classified as ‘CR’ or ‘PR’ by RECIST. The proportion of reduction surgery is defined as the proportion of patients out of all enrolled cases whose actual surgery is reduced compared with the surgery planned at enrolment. The proportion of extended surgery is defined as the proportion of patients out of all enrolled cases whose actual surgery is extended compared with the surgery planned at enrolment. Post-operative limb function is assessed by the International Society of Limb Salvage score. The proportion of limb salvage is defined as the proportion of affected limbs that could be preserved after surgery, with the denominator being all limb-occurring cases enrolled.

### Participant timeline

The participant timeline is summarized in Table 3. Candidates who agree to participate are checked for eligibility based on the inclusion/exclusion criteria, and enrolment is completed after confirmation of eligibility. Physical examination, Eastern Cooperative Oncology Group performance status (PS), body weight, body height, complete blood count (CBC), serum biochemistry, urine analyses, creatinine clearance, chest X-ray and percutaneous saturation of oxygen (SpO_2_) are performed within 14 days before registration. Electrocardiography, computed tomography (CT) of the chest, magnetic resonance imaging (MRI) of the primary site and additional CT/MRI of the associated lymph nodes area are performed within 35 days before registration. Tests of hepatitis and 18-fluorodeoxyglucose positron emission tomography–computed tomography (PET-CT) or bone scintigraphy should also be performed before registration. Participants should commence the treatment protocol within 7 days (arm A) or 28 days (arm B) of registration.

**Table 3:** The study schedule

Arm A	Enrolment	NAC			Surgery		AC		Follow-up
		1	2	3	Before	After	1	2	
Informed consent	○	◯							
Inclusion/exclusion criteria	◯								
Physical examination	○	○	○	○	○	○	○	○	○
Body weight	○	○	○	○	○		○	○	
Height	○								
PS	○	○	○	○	○		○	○	
Blood examinations^a^	○	○	○	○	○		○	○	○
Urine analysis	○		○	○			○	○	
Creatinine clearance	○								
HBs-Ag, HBc-Ab, HBs-Ab, HCV-Ab	○								
Chest X-ray	○		○	○	○		○	○	○
Chest CT	○				○				○
MRI of primary site	○				○				○
CT/MRI for lymph nodes	○								
PET-CT/Bone scintigraphy	○								
SpO_2_	○		○	○			○	○	
ECG	○				○				
Adverse events		○	○	○	○	○	○	○	○
Arm B	Enrolment	Surgery		AC			Follow-up		
		Before	After	1	2	3			
Informed consent	○								
Inclusion/exclusion criteria	◯								
Physical examination	○		○	○	○	○	○		
Body weight	○								
Height	○								
PS	○			○	○	○			
Blood examinations^a^	○	○		○	○	○	○		
Urine analysis	○			○	○	○			
Creatinine clearance	○								
HBs-Ag, HBc-Ab, HBs-Ab, HBC-Ab	○								
Chest X-ray	○			○	○	○	○		
Chest CT	○						○		
MRI of primary site	○						○		
CT/MRI for lymph nodes	○								
PET-CT/Bone scintigraphy	○								
SpO_2_	○			○	○	○			
ECG	○								
Adverse events			○	○	○	○	○		

^a^CBC, serum biochemistry. PS, Performance Status; SpO_2_, percutaneous oxygen saturation; ECG, electrocardiogram.

The criteria for dose reduction or discontinuation are set as follows. In the event of severe myelosuppression, liver dysfunction, diarrhoea, oral mucositis or infection, the dose is reduced by 20% per step in the next course. Further chemotherapy should be terminated if the dose reduction criteria are still met after two steps of dose reduction.

In the event of severe pneumonitis, increased creatinine, arrhythmia, cardiac dysfunction, vertigo, nervous system disorder or haematuria, the chemotherapy should also be discontinued. No other anti-cancer drugs, hormone therapies other than steroids, radiotherapy or immunotherapy should be used during protocol treatment.

In arm A, if any of the following (i–iv) are met after the start of NAC, subsequent chemotherapy should be discontinued: (i) exacerbation of disease after the start of protocol treatment; (ii) the protocol treatment cannot be continued due to adverse events including Grade 4 non-hematologic toxicity and delay of >14 days of the start of the following course; (iii) the patient requests termination of protocol treatment for reasons related to adverse events; and (iv) the patient requests termination of protocol treatment for reasons unrelated to adverse events. In case of discontinuation of NAC, protocol treatment is continued, and surgery is performed after determining tumour reduction efficacy as far as possible. However, AC is not given, and protocol treatment is discontinued upon completion of surgical treatment. Protocol treatment should be discontinued if any of the following (i–vi) are met: (i) progression of disease; (ii) the protocol treatment cannot be continued due to adverse events including Grade 4 non-hematologic toxicity; (iii) the patient requests termination of protocol treatment for reasons related to adverse events; (iv) the patient requests termination of protocol treatment for reasons unrelated to adverse events; (v) death during protocol treatment; and (vi) post-enrolment exacerbation prior to the initiation of protocol treatment (inability to start the protocol treatment due to rapid progression), the discovery of protocol violation, change of treatment due to a change in the pathological diagnosis after enrolment, or other reasons that make the patient ineligible for protocol treatment.

### Sample size

Based on the background provided, the 3-year OS for arms A and B are assumed to be 85%. The non-inferiority margin is 8% in 3-year OS (acceptable HR of 1.61). Assuming 6 years of accrual, 3 years of follow-up, α = 10% (one-sided) and a non-inferiority margin of 1.61, the planned sample size is set at 224 in total and 112 in each arm based on the Schoenfeld & Richter’s method (18). If enrolment is better than expected, the power would be changed from 70% to 75% or 80%.

### Data collection, management, monitoring and auditing

The investigator uses the electronic data capture (EDC) system of the JCOG web entry system to input data into the case report form. Any adverse events are closely monitored by the investigator. Investigators should report severe adverse events to the institutional investigator and study coordinator/study chair and then to the CRB of the National Cancer Center Hospital, as appropriate. The EDC system, Electronic Document Management System (E-DMS) Online (EPS Corporation, Tokyo, Japan), is used for clinical data entry, data management and central monitoring.

Two interim analyses are conducted during the trial to determine whether the primary aim has been achieved: the first interim analysis is conducted during accrual to decide whether or not it is reasonable to continue accrual; the second interim analysis is conducted early after the end of accrual to determine whether to continue follow-up for the planned period. In both cases, if it is determined that the primary aim has been achieved, the study is terminated, and the results of the study are promptly published. In-house monitoring is performed every 6 months by the JCOG Data Center to evaluate the study progress and improve the data quality.

### Statistical analysis

All statistical analyses will be conducted at the JCOG Data Center. The primary analysis will be performed using the stratified Cox proportional hazards model with stratified allocation adjustment factors excluding the centre: histological grade (Grade 2 vs. Grade 3) and T-classification (T2 vs. T3–4). The confidence intervals are constructed with the Wald method. The non-inferiority is confirmed when the upper limit of confidence interval of the HR adjusted for multiplicity is below the margin of 1.61.

To examine the interaction between the treatment effect and the background factors, subgroup analyses will be conducted in the following factors: age, PS, sex, primary site, pathological grading, histological type, tumour size, surgical procedure, surgical margin, post-operative radiation and completion of protocol treatment. Since these analyses are not performed with sufficient power and are not adjusted for multiplicity, results are only to be interpreted as exploratory.

## Discussions

Surgery is the most important treatment for STSs. Chemotherapy has been reported to have an additive effect on OS in high-risk patients and is listed as a consideration in the respective guidelines (16,17). The unsolved current clinical issue is the lack of evidence regarding whether surgery, the most important therapeutic intervention, should be performed first or after chemotherapy for resectable high-risk STS.

If the study has a positive result, the standard treatment for resectable high-risk STSs changes to immediate surgery followed by three courses of AC. It reduces the incidence of extended surgery and amputation of affected limbs and shortens the duration of treatment.

If the result is negative, the standard treatment in Japan remains three courses of NAC followed by surgery and two courses of AC. The result indicates that three courses of AC are inferior to five courses of perioperative chemotherapy. Taken together with the previous report showing that three courses of NAC were non-inferior to five courses of perioperative chemotherapy (8), these are the first results to suggest that the OS benefit of chemotherapy in the neo-adjuvant setting may be greater than in the adjuvant setting for STS.

Whether the results of this trial are positive or negative, they will indicate whether resection or chemotherapy should be performed first.

### Ethics and dissemination

This study is conducted under the ethical principles stipulated in the ‘Declaration of Helsinki’ (revised October 2013) and ‘Clinical Trials Act’ (announced 14 April 2017, enacted 1 April 2018) established by Japan’s Ministry of Health, Labour, and Welfare.

Before enrolment, all patients receive verbal and written information and provide informed consent before enrolment. This study was approved by the National Cancer Center Hospital CRB (CRB3180008) on 30 September 2022 (approval number: T2022003) and registered in the Japan Registry of Clinical Trials (jRCTs031220446).

Any protocol amendment and revision are reviewed by the National Cancer Center Hospital CRB. This study protocol was lately revised in May 2023 (ver. 1.1.0) due to a minor revision. The results of this study will be submitted to international peer-reviewed journals, and key findings will be presented at international scientific conferences.

## Data Availability

All data are incorporated into the article and its online supplementary material.
